# Ultra-Broadband Nonlinearity Enhancement based on a Novel Graphene-Silicon Hybrid Waveguide: Structure Design and Theoretical Analysis

**DOI:** 10.1038/s41598-017-12554-6

**Published:** 2017-09-25

**Authors:** Qiang Jin, Xibin Li, Junfan Chen, Shiming Gao

**Affiliations:** Centre for Optical and Electromagnetic Research, State Key Laboratory of Modern Optical Instrumentation, Zhejiang University, Hangzhou, 310058 China

## Abstract

A graphene-silicon hybrid waveguide with a dielectric spacer is proposed to enhance the nonlinear response in ultra-wide wavelength range by applying graphene’s broadband highly nonlinear optical properties. The chemical potential of the graphene layer is tuned to satisfy the resonance condition and hence a low propagation loss is obtained. The dielectric spacer is used for avoiding additional free-carrier-absorption loss due to carrier interchange between the silicon core and the graphene layer. Aiming at the special waveguide structure with ultra-thin graphene layer, a full-vectorial theoretical model is developed to analyze its nonlinear properties. The waveguide dimensions are optimized in terms of the nonlinear parameter. The proposed hybrid waveguide exhibits high nonlinearity enhancement in an ultra-broad wavelength region covering near-infrared and mid-infrared bands. The conversion efficiency for a degenerate four-wave mixing process reaches −18.5 dB only with a pump power of 0.5 W and a waveguide length of tens of microns. In the wavelength range of 1.3–2.3 μm, the conversion efficiency can be kept stable by adopting suitable waveguide geometry and length. The corresponding 3-dB bandwidth can reach 40–110 nm for each fixed pump. The graphene-silicon hybrid waveguide has the potential to support chip-scale nonlinear applications in both near- and mid-infrared bands.

## Introduction

Graphene, a single layer of carbon atoms arranged in a honeycomb lattice with linear, massless band structure, has attracted great interest by its remarkable electrical and optical properties^1–3^. Despite the two-dimensional and one-atom thick nature of graphene, the complementary metal oxide semiconductor (CMOS)-compatible integration processes in subwavelength dimensions make it a promising candidate for chip-scale optoelectronics. In 2010, the first observation of the coherent nonlinear optical response of graphene at visible and near-infrared frequencies was reported and the exceptionally large third-order susceptibility is ~10^−7^ esu^4^. Since graphene is a film material and the interacting distance is short when light propagates through the film, which will limit the nonlinear effects in graphene. In order to solve this problem, hybrid waveguides considering the composition of graphene and other materials may become ideal choices. In recent years, some kinds of graphene-based photonic devices have been demonstrated ranging from hybrid graphene-silicon waveguide for photodetection^5^ and modulation^6–8^, to graphene-clad fiber for polarization^9^ and wavelength conversion^10^, and to graphene-silicon hybrid optical cavity for regenerative oscillation and four-wave mixing^11^. These hybrid structures can greatly increase the interacting length through the coupling of the evanescent field into the graphene layer. In the past ten years, silicon waveguides has been developed extensively for many nonlinear applications^12^ and exhibit excellent light-confinement ability due to the large refractive index contrast and also high nonlinear response^13^. It will be very attractive to combine graphene and silicon-on-insulator (SOI) nanowire waveguides to form graphene-silicon hybrid waveguides for broadband nonlinear optical purpose.

As we know, a monolayer of pristine graphene exhibits a considerable wavelength-independent absorption (≈ 2.3% per layer) in an ultra-broadband wavelength region involving infrared and visible optical band^14^. In graphene hybrid waveguide structures, the in-plane absorption will become much heavy since the interacting length is greatly lengthened^15^. Large optical loss will affect the efficiency of the nonlinear effects in the waveguide. Fortunately, the absorption of graphene has the chance to be tuned by shifting its electronic Fermi level^16,17^ via electrical gating or chemical doping. As for the broadband nonlinear enhancement of graphene-silicon hybrid waveguides, the dependence of the graphene nonlinearity on the chemical potential and the working wavelength is eager to be explored. The third-order susceptibility of graphene has been experimentally measured in previous contributions^4,18–21^, but their measured results cannot agree well with each other and strongly depend on the light frequency, the measurement method, and the sample preparation. Theoretically, the optical nonlinearity of graphene has been calculated by perturbative treatment through various quantum theories^22–27^. Though their results are quite different, the third-order nonlinearity of graphene is related to these physical condition: chemical potential, realistic scattering rate, and temperature. Based on the single-particle assumption and perturbative calculation, Mikhailov gave a full expression of the third-order conductivity of graphene, which unified the existing theories proposed so far^27^ and provided a clear physical insight of the complex nonlinear optical effects in graphene. Therefore, the loss and nonlinear performance of graphene can be both controlled by suitably setting its chemical potential.

It is quite challengeable to determine the nonlinear wave propagation in the graphene-silicon hybrid waveguide due to the graphene’s one-atom thickness, although the graphene layer can be typically treated as an ultra-thin film with an equivalent bulk nonlinear susceptibility. In conventional waveguides and optical fibers, the linear and nonlinear processes are characterized by using the well-known scalar Helmholtz equation, which is founded on the weak guidance approximation and homogeneous cross-section assumption^28,29^. However, the emerging graphene-silicon hybrid waveguides (i.e. inhomogeneous, high refractive index contrast, and transverse structure incorporating subwavelength features) here exhibit strong guidance with a large longitudinal component of modal fields along the propagation direction, and traditional treatment is not suitable to describe the wave propagation in it accurately. The inhomogeneous and full vectorial solutions of the governing Maxwell’s equation have to be pursued. Without the precondition of weak guidance approximation, a vectorial nonlinear Schrödinger equation for nonlinear pulse propagation has been derived in ideal lossless waveguides with complex transverse structure^30^, which provides a platform for generalizing nonlinear processes like self-phase modulation (SPM), cross-phase modulation (XPM), four-wave-mixing (FWM), Raman and Brillouin scattering. Nevertheless, the impact of optical loss is a non-negligible factor in graphene-silicon hybrid waveguides and will strongly affect its nonlinear efficiency. In particular, we find that, as the wavelength increases, graphene’s imaginary part of the third-order susceptibility may change its sign near the resonance points of photon absorption, thus the loss of graphene cannot be simply treated as either two-photon absorption (TPA) or absorption saturation.

In this paper, we propose a geometry-optimized graphene-silicon hybrid waveguide with a dielectric spacer for optical nonlinear enhancement at near- and mid-infrared frequencies. The dielectric spacer is used to block the carrier exchange and hence reduce the additional loss. The full vectorial model is developed for the analysis of the nonlinear continuous wave propagation by presenting an effective nonlinear parameter that combines the nonlinear phase shift term and the nonlinear loss term. Numerical results show the graphene-silicon hybrid waveguide has an ultra-highly nonlinear response. The degenerated FWM (DFWM) effect in the proposed graphene-silicon hybrid waveguide shows broadband performance and the nonlinear enhancement can cover both the near- and mid-infrared bands.

**Figure 1:**
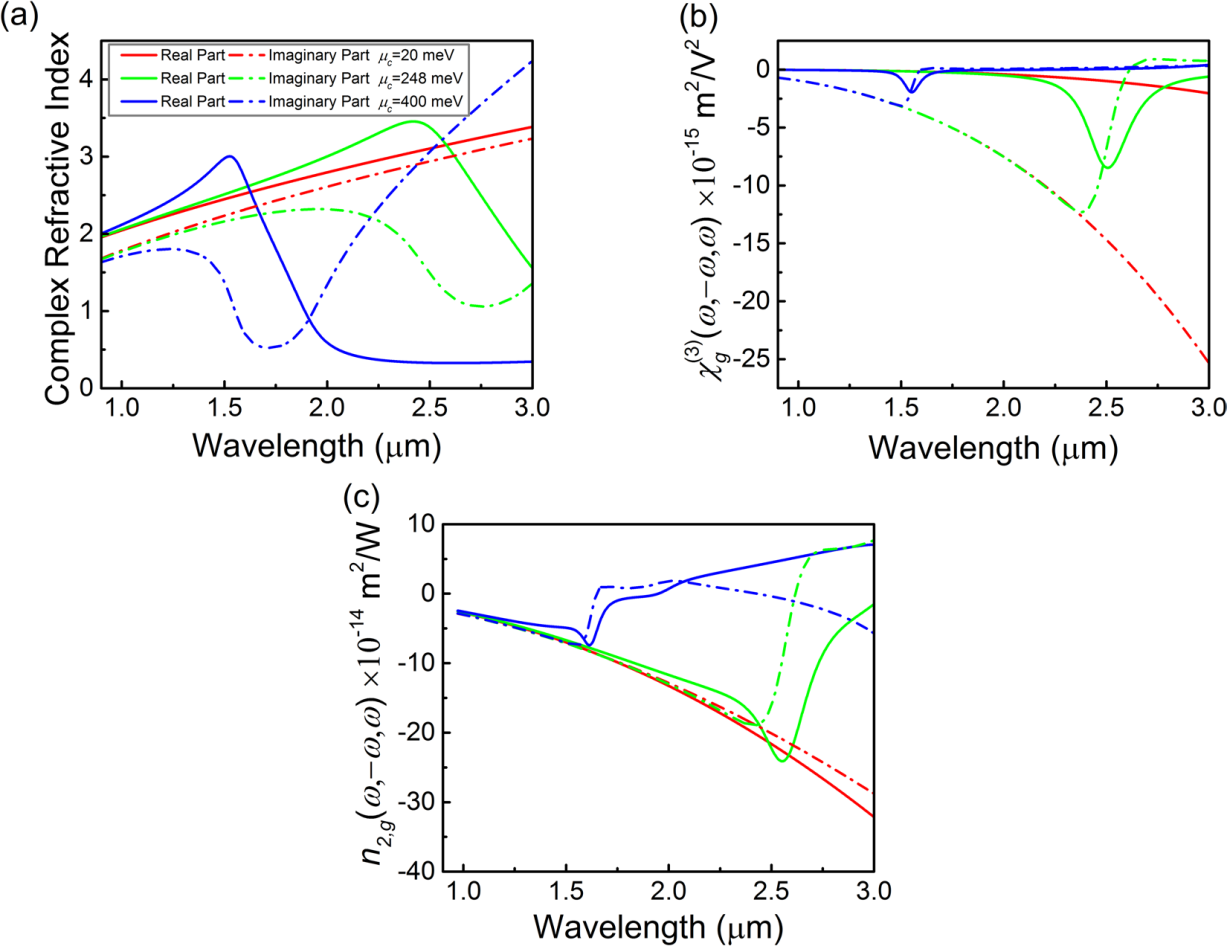
Linear and nonlinear optical properties of graphene. The physical condition for the calculation of both linear and third order conductivity of graphene is: the intraband and interband relaxation parameters $\hslash {{\rm{\Gamma }}}_{{\bf{i}}}=\hslash {{\rm{\Gamma }}}_{e}=$ 33 meV, and the thickness of graphene is assumed to be 0.5 nm. (**a**) Complex refractive index; (**b**) complex third-order susceptibility; (**c**) complex Kerr coefficients at various chemical potentials.

Graphene’s complex surface conductivity consists of intraband and interband parts and depends on the parameters including the radian frequency *ω*, relaxation parameter *Γ*, temperature *T*, and chemical potential ${\mu }_{c}$ ^31,32^. In the linear optical response, the equivalent complex relative permittivity of the Δ-thick graphene can be expressed as ${\varepsilon }_{g}=1+i{\sigma }_{g}^{(1)}/({\varepsilon }_{0}\omega {\rm{\Delta }})$, where the linear conductivity ${\sigma }_{g}^{(1)}$ is calculated from the Kubo formula^33^. At optical frequencies, graphene’s complex refractive index ${\tilde{n}}_{g}={n}_{g}+i{\kappa }_{g}$ is defined as ${\tilde{n}}_{g}=\sqrt{{\varepsilon }_{g}}$, where ${\kappa }_{g}$ is called the extinction coefficient and determines the linear optical absorption. As shown in Fig. 1a, for $\hslash \omega \,\gg \,2|{\mu }_{c}|$, the attenuation constant is very large and vary slightly at high optical frequency. When the photon energy drops below the transition threshold ($\hslash \omega  < 2|{\mu }_{c}|$), no interband transition is allowed and the absorption sharply decreases. However, in the long wavelength range, the intraband transition becomes dominant and the absorption becomes obvious once more. The location of this low absorption window depends on graphene’s chemical potential and can be tuned in a wide wavelength range.

As for the nonlinear case, we only consider the third-order conductivity ${\sigma }_{g}^{(3)}({\omega }_{1},\,{\omega }_{2},\,{\omega }_{3})$ and neglect higher order nonlinearities due to the inversion symmetry of the graphene lattice. According to the analytic results of the third-order conductivity (such as Kerr nonlinearity in Fig. 9a in ref.^27^), for a given chemical potential there are certain frequency ranges where the nonlinear response of graphene are exceptionally large due to the resonance enhancement of photon absorption. At high (infrared) frequencies, the imaginary part of third-order conductivity $\text{Im}({\sigma }_{g}^{(3)}(\omega ,\,-\omega ,\,\omega ))$ of Kerr nonlinearity achieves its maximum at the resonant condition: $\hslash \omega \approx 2|{\mu }_{c}|$. As the phenomenological relaxation parameter *Γ* is a small value ([0, 60] meV)^24^, the position of high-frequency resonance mainly depends on the Fermi level, i.e. ${\mu }_{c}$. The equivalent complex third-order susceptibility ${\chi }_{g}^{(3)}(\omega ,\,-\omega ,\,\omega )$ of Kerr nonlinearity relates to the third-order conductivity as ${\chi }_{g}^{(3)}(\omega ,\,-\omega ,\,\omega )={\sigma }_{g}^{(3)}(\omega ,\,-\omega ,\,\omega )/(-i{\varepsilon }_{0}\omega {\rm{\Delta }})$. Based on the analytical theory in ref.^27^, in a broad band (0.9 ~ 3 μm), Fig. 1b shows the inverted hump-shaped response of ${\chi }_{g}^{(3)}$ at different chemical potentials. However, assuming a low chemical potential $|{\mu }_{c}|=0.1$ eV, the calculated values of $\mathrm{Re}({\chi }_{g}^{(3)})$ are smaller than the results reported by Hendry *et al*.^4^ by two orders of magnitude and have weak dependence on the relaxation parameter in the range of [0, 60] meV. In practice, we adopt the nonlinear refractive index ${n}_{2}$ rather than the third-order susceptibility to characterize the Kerr nonlinearity of a particular kind of material, which relates to the real part of ${\chi }^{(3)}$ as ${n}_{2}=3/(4{\boldsymbol{c}}\varepsilon )Re({\chi }^{(3)})$ (*ε* denotes the material’s permittivity). As both graphene’s linear and nonlinear conductivities have considerable real parts and imaginary parts, the derivation of nonlinear refractive index ${n}_{2,g}$ should follow the results of del Corso and Soles^34^. For a given chemical potential, the complex ${n}_{2,g}$ of grapheme depicted in Fig. 1c has different shape from the real part of ${\chi }_{g}^{(3)}$ in Fig. 1b, and both the real and imaginary parts achieve their maxima near the resonant frequency ($\hslash \omega \approx 2|{\mu }_{c}|$). Usually, the positive imaginary part of ${n}_{2}$ indicates the TPA process. While in graphene, the imaginary part is negative for $\hslash \omega  > 2|{\mu }_{c}|$ and can be understood as a correction to the simple linear prediction of the absorption as the one-photon absorption is always positive in this frequency range. We also find that the calculated values of $\mathrm{Re}({n}_{2,g})$ at specific wavelength is in agreement with the experimental measurements^21^. For a monolayer of pristine graphene with ${\mu }_{c}=0.02$ eV (taking into the slight substrate-induced doping), the nonlinear refractive index (red curve in Fig. 1c) is in the order of 10^−13 ^∼ 10^−12^ and essentially dispersionless over the broad wavelength range. By tuning graphene’s Fermi level, the peak response of $|\mathrm{Re}({n}_{2,g})|$ can be shifted closely to the central frequency ${\omega }_{0}$ of the interested frequency band according to the resonance condition $\hslash {\omega }_{0}\approx 2|{\mu }_{c}|$. In the vicinity of the most important feature at $\hslash {\omega }_{0}\approx 2|{\mu }_{c}|$, the amplitude of $|\mathrm{Re}({n}_{2,g})|$ in Fig. 1c shows no obvious increase compared with the pristine grapheme (${\mu }_{c}=0.02$ eV), but the linear absorption is greatly reduced as shown in Fig. 1a. Therefore, shifting the Fermi level to match the resonance condition at ${\omega }_{0}$ shows the potential in improving graphene’s nonlinear performance by increasing the figure of merit (FOM)^10^.

**Figure 2:**
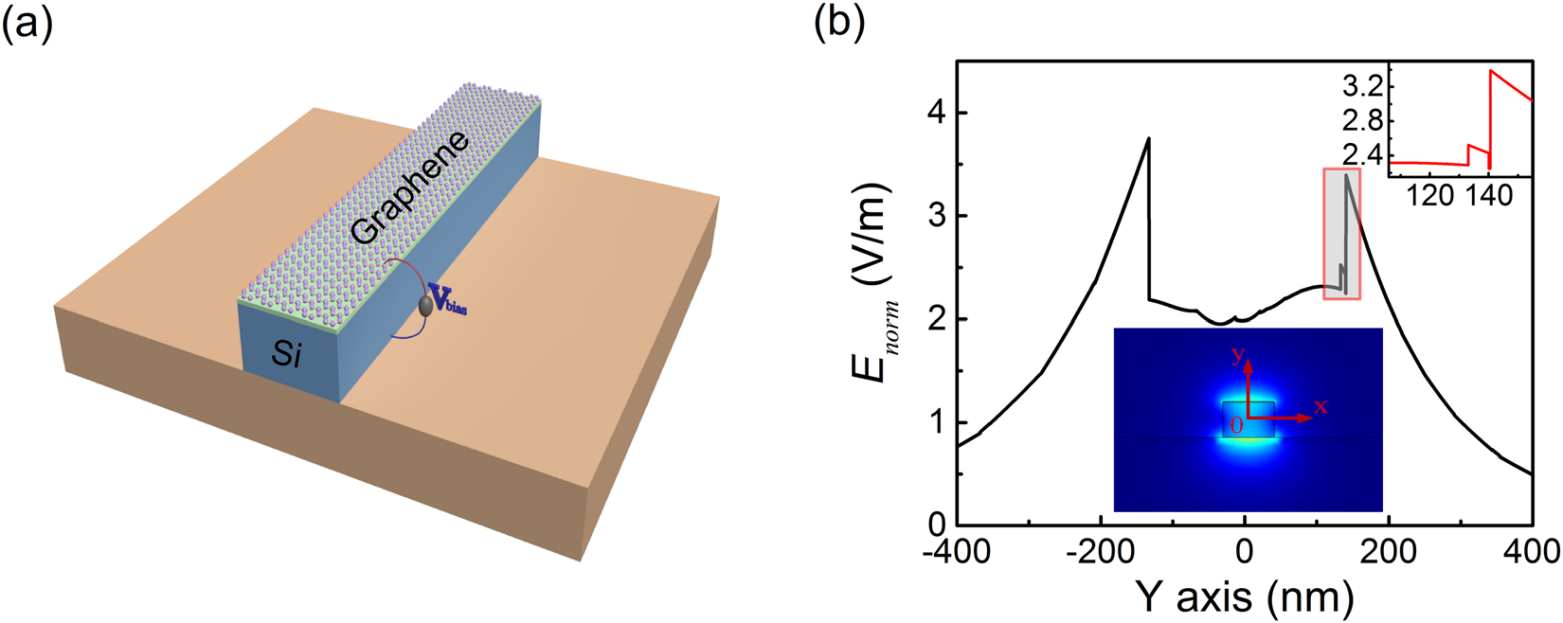
graphene-silicon hybrid waveguide. (**a**) Three-dimensional schematic illustration of the graphene-silicon hybrid waveguide. (**b**) The normalized electric field distribution along the y axis at 1.55 μm with graphene’s chemical potential ${\mu }_{c}=0.4$ eV. Insets: top right corner, zoom-in details near the graphene region; bottom, the optical TM mode.

Figure 2a shows the structure of the proposed graphene-silicon hybrid waveguide. Monolayer graphene film is on the top of the SOI nanowire and between them there is a 7-nm thick Al_2_O_3_ spacer^6^. This insulating layer blocks the carrier interchange between the silicon core and the graphene layer as the photoexcited electrons may transfer from graphene to silicon and cause additional free carrier absorption^35^. Then a gate voltage can be applied to this hybrid waveguide for actively tuning the Fermi level of the cladding graphene film. Further considering the effective interaction between the light field and the graphene in the particular band centered at ${\omega }_{0}$, the dimensions of the waveguide cross-section is designed and optimized to support the electric field maximized at its top and bottom surfaces (fundamental TM mode) when graphene’s Fermi level is tuned to match the resonant condition $\hslash {\omega }_{0}\approx 2|{\mu }_{c}|$. Using the finite-element-frequency-domain (FEFD) method, the electric field distribution at 1.55 μm for a waveguide cross-section of 360 nm × 266 nm is simulated in the inset of Fig. 2b. Also, Fig. 2b shows the normalized electric field distribution ${E}_{norm}$ along the y axis and sufficient light concentration can be expected near the graphene-silicon boundary. As the fact that graphene only interacts with the tangential (in-plane) electric field of electromagnetic waves, we only consider the tangential components of the electric field and magnetic field in the graphene layer in the following simulation.

In order to derive the coupled equations for parametric conversion process in graphene-silicon hybrid waveguides, we start from the conjugated form of the Lorentz reciprocity theorem in frequency domain: (see e.g. ref.^36^)

1 $${\int }_{s}\nabla \cdot {{\bf{F}}}_{c}ds=\frac{\partial }{\partial z}{\int }_{s}{{\bf{F}}}_{c}\cdot \hat{z}ds+{\oint }_{l}{{\bf{F}}}_{c}\cdot \hat{n}dl$$

The arbitrary integration area *s* is in the waveguide cross-section plane and $\hat{z}$ is the unit vector oriented along the longitudinal axis. At a certain frequency *ω*, we introduce two sets of electromagnetic fields: the vectors (${{\bf{E}}}_{0}(\omega )$, ${{\bf{H}}}_{0}(\omega )$) represent the total unperturbed fields of the ideal lossless waveguide and the vectors (${\bf{E}}(\omega )$, ${\bf{H}}(\omega )$) correspond to the situation under which the optical properties are perturbed both by the nonlinear optical effects, such as SPM, XPM, and FWM, and the linear and nonlinear losses. They are related to the vector ${{\bf{F}}}_{c}$, which is defined as ${{\bf{F}}}_{c}={{\bf{E}}}_{0}\times {{\bf{H}}}^{\ast }+{{\bf{E}}}^{\ast }\times {{\bf{H}}}_{0}$. And if the integral area *s* extends to an infinite distance, equation (1) can be simplified as $\,{\int }_{\infty }\nabla \cdot {{\bf{F}}}_{c}ds=\frac{\partial }{\partial z}{\int }_{\infty }{{\bf{F}}}_{c}\cdot \hat{z}ds$. Using Maxwell’s equations, the left side of equation (1) can be written as $-i\omega {\int }_{\infty }\delta {{\bf{P}}}^{\ast }(\omega )\cdot {{\bf{E}}}_{0}(\omega )ds$, where $\delta {\bf{P}}(\omega )=\delta \varepsilon {\bf{E}}(\omega )\equiv \delta {{\bf{P}}}_{lin}(\omega )+{{\bf{P}}}_{nl}(\omega )$ is the perturbed polarization with $\delta {{\bf{P}}}_{lin}(\omega )$ and ${{\bf{P}}}_{nl}(\omega )$ representing its linear and nonlinear components, respectively. The change of the permittivity $\delta \varepsilon (\omega )$, induced by both linear effects (such as optical losses and free-carrier generation) and nonlinear effects, is the origination of this perturbed polarization^37^.

Supposing the practical field for graphene-silicon hybrid waveguides consists of a superposition of quasi-monochromatic waves, with $\omega ^{\prime}$ denoting any one of the frequencies involved, we can write the total electric field ${\bf{E}}(t)$ and its Fourier transform ${\bf{E}}(\omega )$ as

2 $${\bf{E}}(t)=\frac{1}{2}\sum _{\omega ^{\prime}  > 0}[{{\bf{E}}}_{\omega ^{\prime} }\exp (-i\omega ^{\prime} t)+{{\bf{E}}}_{-\omega ^{\prime} }\exp (i\omega ^{\prime} t)]$$

3 $${\bf{E}}(\omega )=\frac{1}{2}\sum _{\omega ^{\prime}  > 0}[{{\bf{E}}}_{\omega ^{\prime} }\delta (\omega -\omega ^{\prime} )+{{\bf{E}}}_{-\omega ^{\prime} }\delta (\omega +\omega ^{\prime} )]$$

where the monochromatic-wave amplitude ${{\bf{E}}}_{\omega ^{\prime} }$ is a vector phasor and ${{\bf{E}}}_{-\omega ^{\prime} }={{{\bf{E}}}_{\omega ^{\prime} }}^{\ast }$ for ${\bf{E}}(t)$ is real.

The vectorial solution of Maxwell’s equations for the unperturbed fields in such ideal lossless waveguide results in a complete orthonormal set of forward, backward, and radiation propagation modes with certain boundary conditions applied^36^. In our proposed single-mode hybrid waveguides, the input light is coupled into one particular polarization of the fundamental mode, like the TM mode, and one can see no coupling between these two polarization modes during the light propagation by simply applying the reciprocity theorem above. Thus for the propagating TM mode, the forward term of the unperturbed fields notated in vector phasor form (evaluated at $\omega ^{\prime}$) can be expressed as

4 $${{\bf{E}}}_{0{\omega }^{{\rm{^{\prime} }}}}=\frac{{\bf{e}}(x,y,{\omega }^{{\rm{^{\prime} }}})}{\sqrt{N}}{e}^{i\beta z}\,;\quad {{\bf{H}}}_{0{\omega }^{{\rm{^{\prime} }}}}=\frac{{\bf{h}}(x,y,{\omega }^{{\rm{^{\prime} }}})}{\sqrt{N}}{e}^{i\beta z}$$

where $N=\frac{1}{2}\mathrm{Re}({\int }_{\infty }{\bf{e}}(x,y,\omega ^{\prime} )\times {{\bf{h}}}^{\ast }(x,y,\omega ^{\prime} )\cdot \hat{z}ds)$ is a normalization constant that defines the mode power and *β* is the propagation constant at frequency $\omega ^{\prime}$. Modal field distributions, the vector phasors ${\bf{e}}(x,y,\omega ^{\prime} )$ and ${\bf{h}}(x,y,\omega ^{\prime} )$, of propagating modes in the graphene-silicon hybrid waveguide can be obtained through a variety of numerical methods including the finite element method. Assuming that the linear and nonlinear perturbations are small enough not to affect the modal field distribution, the corresponding perturbed fields ${\bf{E}}{}_{\omega ^{\prime} }$ and ${{\bf{H}}}_{\omega ^{\prime} }$ can be expanded according to the unperturbed ones:

5 $${{\bf{E}}}_{{\omega }^{{\rm{^{\prime} }}}}=a(z,{\omega }^{{\rm{^{\prime} }}})\frac{{\bf{e}}(x,y,{\omega }^{{\rm{^{\prime} }}})}{\sqrt{N}}{e}^{i\beta z}\,;\quad {{\bf{H}}}_{{\omega }^{{\rm{^{\prime} }}}}=a(z,{\omega }^{{\rm{^{\prime} }}})\frac{{\bf{h}}(x,y,{\omega }^{{\rm{^{\prime} }}})}{\sqrt{N}}{e}^{i\beta z}$$

It should be noted that the frequency dependence of perturbed fields is totally contained within the amplitude coefficient $a(z,\omega ^{\prime} )$, and the mode optical power is $P(\omega ^{\prime} )=a{(z,\omega ^{\prime} )}^{2}$. We only consider the unidirectional wave propagation for which the back scattering of a forward propagating laser beam is neglected, and hence ignore the coupling between the unperturbed field with the backward and radiation modes of the perturbed field^30^. Substituting equations (2)–(5) into the simplified form of equation (1), one can obtain:

6 $$\begin{array}{c}\sum _{\omega ^{\prime}  > 0}\frac{\partial }{\partial z}[a(z,\omega ^{\prime} )\delta (\omega -\omega ^{\prime} )+a(z,-\omega ^{\prime} )\delta (\omega +\omega ^{\prime} )]\\ =\sum _{\omega ^{\prime}  > 0}\frac{i\omega {e}^{-i\beta z}}{2\sqrt{N}}{\int }_{\infty }({{\bf{e}}}^{\ast }(x,y,\omega ^{\prime} )\delta (\omega -\omega ^{\prime} )+{{\bf{e}}}^{\ast }(x,y,-\omega ^{\prime} )\delta (\omega +\omega ^{\prime} ))\cdot \delta {\bf{P}}(x,y,\omega )ds\end{array}$$

In frequency domain, the induced *n* ^th^-order polarization can be written in a general form:

7 $${{\bf{P}}}^{({\rm{n}})}(\omega )={\varepsilon }_{0}{\int }_{-\infty }^{+\infty }d{\omega }_{1}\cdots {\int }_{-\infty }^{+\infty }d{\omega }_{n}{\chi }^{({\rm{n}})}(-{\omega }_{\sigma };{\omega }_{1},\ldots ,{\omega }_{n})|{\bf{E}}({\omega }_{1})\cdots {\bf{E}}({\omega }_{n})\delta (\omega -{\omega }_{\sigma })$$

Equation (7) shows a complete description that requires the knowledge of the tensorial and dispersive properties of the nonlinear susceptibility ${\chi }^{(n)}$. Since there is no second-order nonlinearity in graphene or silicon, here we only consider the third-order nonlinear processes and neglect higher order nonlinearities, i.e. ${{\bf{P}}}_{nl}(\omega )\approx {{\bf{P}}}^{(3)}(\omega )$. As silicon belongs to the m3m point-group symmetry^28^, in Cartesian coordinates, among 81 elements of ${\chi }_{ijkl}^{(3)}$ (*i, j, k, l = x, y, z*) only 21 elements are nonzero, which depend on only four independent quantities (${\chi }_{xxxx}^{(3)},\,{\chi }_{xyxy}^{(3)},\,{\chi }_{xyyx}^{(3)},\,{\chi }_{xxyy}^{(3)}$). In practice, the most relevant nonlinearity is the Kerr nonlinearity involving only one frequency, i.e. ${\chi }_{ijkl}^{(3)}(-\omega ;\omega ,-\omega ,\omega )$, and in the wavelength range considered, photon energy is well below silicon’s energy gap ${E}_{g,Si}$(≈1.12 eV). Therefore, the last three terms are equal. As a result, ${\chi }_{ijkl}^{(3)}$ can be written as:

8 $${\chi }_{ijkl}^{(3)}={\chi }_{xxxx}^{(3)}[\frac{\rho }{3}({\delta }_{ij}{\delta }_{kl}+{\delta }_{ik}\delta {}_{jl}+{\delta }_{il}{\delta }_{jk})+(1-\rho ){\delta }_{ijkl}]$$

where $\rho \equiv 3{\chi }_{xxyy}^{(3)}/{\chi }_{xxxx}^{(3)}$ represents the nonlinearity anisotropy and has a nearly constant real value for $\hslash \omega  < {E}_{g,Si}$. Within the rest of this paper we ignore the last term of equation (8) which affects the polarization dependence of nonlinear effects in silicon waveguides and thus *ρ* = 1^30^. The real and imaginary parts of ${\chi }_{xxxx}^{(3)}$ can be derived from the Kerr coefficient and the TPA coefficient^28^, so here its wavelength dependence is determined by the experimental measurements^38^ through the polynomial fit method. In the following numerical calculation, silicon’s third-order susceptibilities for the corresponding SPM, XPM and FWM processes are the same and derived from the experimental Kerr nonlinearity. Due to the two-dimensional nature of graphene^22^, among all eight nonzero components of the third-order susceptibility there are three are independent: $\,{\chi }_{xxxx}^{(3)}={\chi }_{xxzz}^{(3)}+{\chi }_{xzxz}^{(3)}+{\chi }_{xzzx}^{(3)}$. Recent research reports have established an initial general theory of third-order nonlinear electrodynamic effects of graphene under some conditions, such as linear-dispersion band structure approximation, two-band tight-binding, single-particle effect and so on^23,27^. Beneath the present theory framework, it is reasonable to treat the three independent components of graphene’s nonlinearity as equal.

For the continuous-wave DFWM process, the total propagation field is the superposition of the monochromatic waves with frequencies of ${\omega }_{p}$, ${\omega }_{s}$, and ${\omega }_{i}$, where ${\omega }_{i}=2{\omega }_{p}-{\omega }_{s}$. Here the labels *p*, *s*, and *i* stands for the pump light, the signal light, and the idler light, respectively. In this case, the nonlinear term for the converted idler lightwave ${\omega }_{i}$ on the right side of equation (6) can be written as^30^:

9 $$\begin{array}{rcl}\frac{i{\omega }_{i}{e}^{-i{\beta }_{i}z}}{2\sqrt{{N}_{i}}}{\int }_{\infty }{{\bf{e}}}_{i}^{\ast }\cdot {{\bf{P}}}_{nl}(x,y,{\omega }_{i})ds & = & \frac{3i{\omega }_{i}}{16}{\varepsilon }_{0}{\int }_{\infty }[{\chi }_{{xxxx}}^{(3)}(-{\omega }_{i};{\omega }_{p},{\omega }_{p},-{\omega }_{s})\\  &  & \times \frac{{a}_{p}^{2}{a}_{s}^{\ast }}{3\sqrt{{N}_{i}{{N}_{p}}^{2}{N}_{s}}}(2({{\bf{e}}}_{p}\cdot {{\bf{e}}}_{s}^{\ast })({{\bf{e}}}_{i}^{\ast }\cdot {{\bf{e}}}_{p})+({{\bf{e}}}_{p}\cdot {{\bf{e}}}_{p})({{\bf{e}}}_{i}^{\ast }\cdot {{\bf{e}}}_{s}^{\ast })){e}^{-i({\beta }_{i}+{\beta }_{s}-2{\beta }_{p})z}\\  &  & +\,{\chi }_{{xxxx}}^{(3)}(-{\omega }_{i};{\omega }_{p},{\omega }_{p},-{\omega }_{i})\frac{2{|{a}_{p}|}^{2}{a}_{i}}{3{N}_{i}{N}_{p}}({|{{\bf{e}}}_{p}|}^{2}{|{{\bf{e}}}_{i}|}^{2}+{|{{\bf{e}}}_{i}\cdot {{\bf{e}}}_{p}^{\ast }|}^{2}+{|{{\bf{e}}}_{i}\cdot {{\bf{e}}}_{p}|}^{2})\\  &  & +\,{\chi }_{{xxxx}}^{(3)}(-{\omega }_{i};{\omega }_{s},{\omega }_{s},-{\omega }_{i})\frac{2{|{a}_{s}|}^{2}{a}_{i}}{3{N}_{i}{N}_{s}}({|{{\bf{e}}}_{s}|}^{2}{|{{\bf{e}}}_{i}|}^{2}+{|{{\bf{e}}}_{i}\cdot {{\bf{e}}}_{s}^{\ast }|}^{2}+{|{{\bf{e}}}_{i}\cdot {{\bf{e}}}_{s}|}^{2})\\  &  & +\,{\chi }_{{xxxx}}^{(3)}(-{\omega }_{i};{\omega }_{i},{\omega }_{i},-{\omega }_{s})\frac{{|{a}_{i}|}^{2}{a}_{i}}{3{N}_{i}^{2}}(2{|{{\bf{e}}}_{i}|}^{4}+{|{{\bf{e}}}_{i}^{2}|}^{2})]ds\\  & = & i{\gamma }_{ipps}{a}_{p}^{2}{a}_{s}^{\ast }{e}^{-i({\beta }_{i}+{\beta }_{s}-2{\beta }_{p})z}+i2{\gamma }_{ippi}{|{a}_{p}|}^{2}{a}_{i}+i2{\gamma }_{issi}{|{a}_{s}|}^{2}{a}_{i}+i{\gamma }_{iiii}{|{a}_{i}|}^{2}{a}_{i}\end{array}$$

The electric/magnetic-field-related terms in equation (9), different from that of the scalar form^28^, are the direct results of full vectorial calculation. After integrating them over the waveguide cross-section plane with the spatial distribution of the third-order nonlinearity as its weighting factor, one can get the nonlinear parameters: ${\gamma }_{ipps}$ describes the four-wave mixing process, ${\gamma }_{ippi}$ and ${\gamma }_{issi}$ are responsible for XPM and ${\gamma }_{iiii}$ relates to SPM. Their real parts characterize the capability of attaining nonlinear phase-shift during the light propagation, while the imaginary parts play an important role in the phenomena of nonlinear loss (TPA and light absorption saturation).

The linear part of the perturbed polarization $\,\delta {{\bf{P}}}_{lin}$ in equation (6) is determined by the linear change in the dielectric constant induced by optical losses in silicon and graphene and free carrier generation in silicon core. In the wavelength range considered here, the photon energy $\hslash \omega$ is below the band gap ${E}_{g,Si}$ of silicon, so the interband one-photon absorption (1PA) in the silicon core is forbidden. While for the case of Fermi-level-tunable graphene, the linear absorption coefficient can be calculated from its complex surface conductivity as stated above. Using the finite element method, the intrinsic linear absorption coefficient of this graphene-silicon hybrid waveguide can be derived from the imaginary part of its effective mode index. Besides the intrinsic linear absorption coefficient in the hybrid materials, the total linear loss coefficient ${\alpha }_{lin}$ also includes an empirically additional loss due to the waveguide fabrication such as the sidewall roughness, which is assumed to be 3 dB/cm. When $\hslash \omega  > {E}_{g,Si}/2$, TPA occurs in silicon and the interband transition will generate free carriers, which will cause free carrier absorption ${\alpha }_{F}$ and affect silicon’s refractive index. In practice, it’s common to employ the empirical formulas (18)–(20) in ref.^28^ to calculate these factors^39^. We find that, compared with the absorption coefficient, the variation of the effective mode index is negligible. As stated above, it’s reasonable to ignore higher-order multiphoton absorption under propagation of weak light field. Thus, in graphene-silicon hybrid waveguide, the linear term of equation (6) can be expressed as:

10 $$\frac{i{\omega }_{i}{e}^{-i{\beta }_{i}z}}{2\sqrt{{N}_{i}}}{\int }_{\infty }{{\bf{e}}}_{i}^{\ast }\cdot {{\bf{P}}}_{lin}(x,y,{\omega }_{i})ds=-\frac{1}{2}({\alpha }_{lin}({\omega }_{i})+{\alpha }_{F}({\omega }_{i})){a}_{i}$$

With all necessary parameters settled, the coupled equations of the amplitude evolution for the interacting frequencies (${\omega }_{i},\,{\omega }_{s},\,{\omega }_{p}$) are obtained from equation (6):

11 $$\frac{\partial }{\partial z}{a}_{i}\,=-\frac{1}{2}[{\alpha }_{lin}({\omega }_{i})+{\alpha }_{F}({\omega }_{i})]{a}_{i}+i{\gamma }_{iiii}{|{a}_{i}|}^{2}{a}_{i}+i2{\gamma }_{ippi}{|{a}_{p}|}^{2}{a}_{i}+i2{\gamma }_{issi}{|{a}_{s}|}^{2}{a}_{i}+i{\gamma }_{ipps}{a}_{p}^{2}{a}_{s}^{\ast }{e}^{-i({\beta }_{i}+{\beta }_{s}-2{\beta }_{p})z}$$

12 $$\frac{\partial }{\partial z}{a}_{s}=-\frac{1}{2}[{\alpha }_{lin}({\omega }_{s})+{\alpha }_{F}({\omega }_{s})]{a}_{s}+i{\gamma }_{ssss}{|{a}_{s}|}^{2}{a}_{s}+i2{\gamma }_{spps}{|{a}_{p}|}^{2}{a}_{s}+i2{\gamma }_{siis}{|{a}_{i}|}^{2}{a}_{s}+i{\gamma }_{sppi}{a}_{p}^{2}{a}_{i}^{\ast }{e}^{-i({\beta }_{i}+{\beta }_{s}-2{\beta }_{p})z}$$

13 $$\frac{\partial }{\partial z}{a}_{p}=-\frac{1}{2}[{\alpha }_{lin}({\omega }_{p})+{\alpha }_{F}({\omega }_{p})]{a}_{p}+i{\gamma }_{pppp}{|{a}_{p}|}^{2}{a}_{p}+i2{\gamma }_{piip}{|{a}_{i}|}^{2}{a}_{p}+i2{\gamma }_{pssp}{|{a}_{s}|}^{2}{a}_{p}+i2{\gamma }_{pips}{a}_{i}{a}_{s}{a}_{p}^{\ast }{e}^{i({\beta }_{i}+{\beta }_{s}-2{\beta }_{p})z}$$

The above system of coupled equations are general and can be applied to an arbitrary waveguide. In the following, we will numerically investigate the wave dynamics upon propagation in the proposed graphene-silicon hybrid waveguide and discuss its nonlinear performance (nonlinear parameter, conversion efficiency and conversion bandwidth) in the near- and mid-infrared wavelength range.

From the derivation above, all the effective parameters of the coupled equations can be considered as the optical properties of the corresponding material averaged over an inhomogeneous cross-section weighted by the field distribution. In order to achieve the best nonlinear performance of a DFWM process in the graphene-silicon hybrid waveguide, the cross section dimensions should be designed to maximize the nonlinear parameter at the pump wavelength ${\lambda }_{p}$, and then the waveguide length and pump power should be optimized for the highest conversion efficiency. According to equation (9), the effective nonlinear parameter $\gamma$ for Kerr nonlinearity at frequency $\omega$ can be written as

14 $$\gamma (\omega )=\frac{\omega {\varepsilon }_{0}{\int }_{\infty }{\chi }_{{xxxx}}^{(3)}(-\omega ;\,\omega ,\,\omega ,\,-\omega )(2{|{\bf{e}}|}^{4}+|{{\bf{e}}}^{2}{|}^{2})ds}{{\rm{16}}{N}^{2}}$$

**Figure 3:**
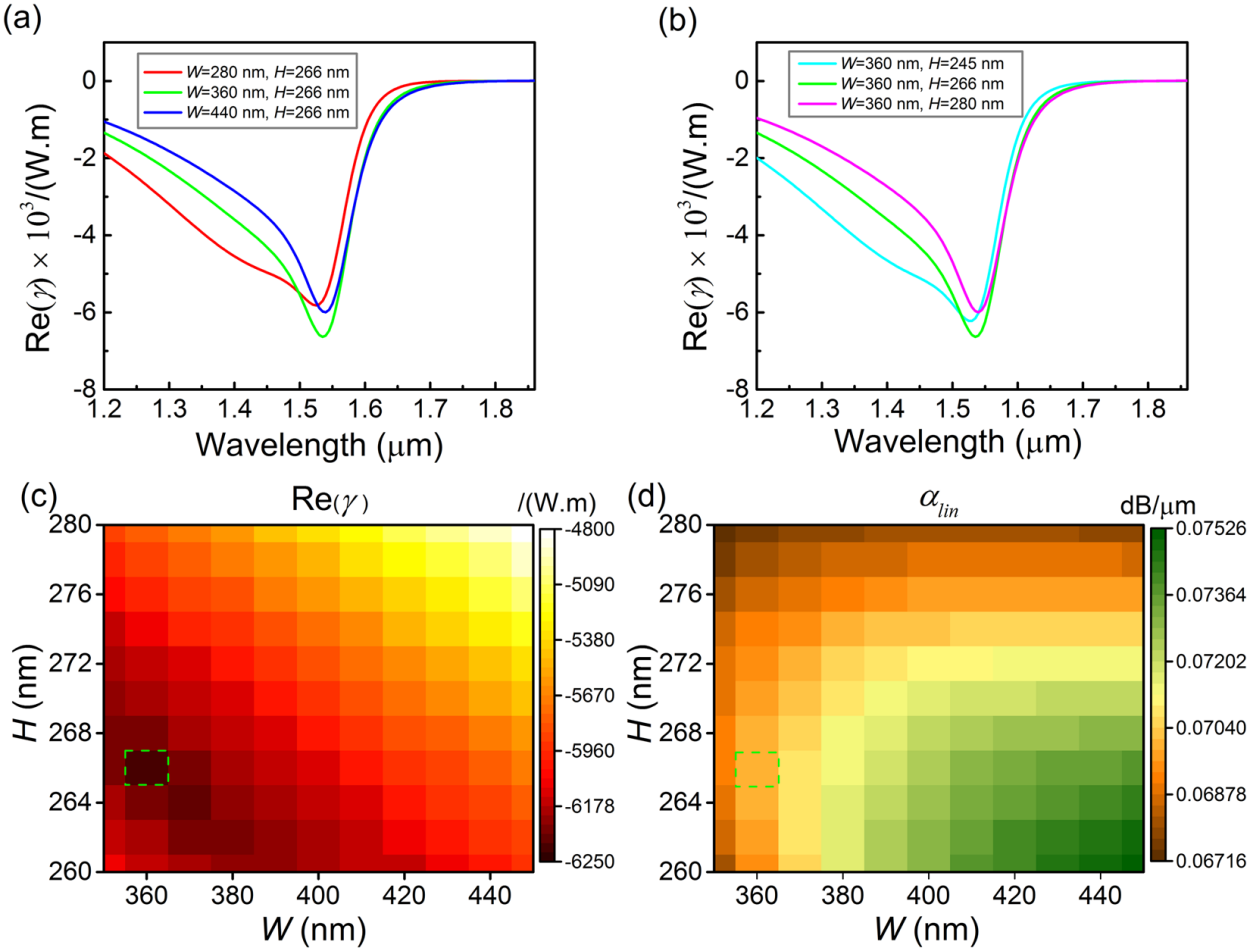
Optimization of the cross-sectional dimensions. Graphene’s Fermi level is set at 0.4 eV. The calculated $\mathrm{Re}(\gamma )$ curves in the band of 1.20–1.86 μm when the waveguide’s (**a**) width or (**b**) height varies. (**c**) The distribution of $\mathrm{Re}(\gamma )$ when sweeping waveguide’s width and height simultaneously. The green dotted bordered rectangle marks the optimal dimensions (360 nm × 266 nm) when the pump wavelength is 1.55 μm. (**d**) The corresponding linear loss of the graohene-silicon hybrid waveguide.

During the optimization, we take the telecom band as an example and set the pump wavelength ${\lambda }_{p}$ at 1.55 μm. Though graphene and silicon have shown their broadband nature of both linear and nonlinear properties in near- and mid-infrared spectrum, for a specified sectional dimension of the graphene-silicon hybrid waveguide, simulation results show that $\mathrm{Re}(\gamma )$ has a smooth inverted hump-shaped distribution with respect to the light wavelength. The Fermi level of graphene satisfy the resonant condition $\hslash {\omega }_{p}\approx 2|{\mu }_{c}|$ for the pump at 1.55 μm, i.e. $|{\mu }_{c}|=0.4$ eV, and as depictedin Fig. 3a and b, the position of the peak $\mathrm{Re}(|\gamma |)$ depends on the dimensions of waveguide’s cross-section and will shift to longer/shorter wavelength as the width *W* (height *H*) increases/decreases. So the bandwidth of nonlinear response is limited by the wavelength-selected performance of the waveguide structure itself. Therefore, the best nonlinear parameter in the wavelength band interested can be achieved through: firstly, tuning the Fermi level of graphene to match the resonant condition at the pump wavelength ${\lambda }_{p}$, and secondly, sweeping the waveguide’s width and height simultaneously to get the maximum $|\gamma |$ at ${\lambda }_{p}$. Under this determined chemical potential for the pump at 1.55 μm, Fig. 3c simulates the nonlinear parameter as a function of the waveguide width and height. The hybrid waveguide is optimized to be 360 nm × 266 nm at 1.55 μm to acquire the maximum absolute nonlinear parameter of 6246.9 W^−1^m^−1^, an order of magnitude larger than that in graphene-silicon hybrid cavity^11^. The waveguide’s linear loss ${\alpha }_{lin}$ is given in Fig. 3d, where one can find that the linear loss is relatively low for the optimized waveguide and the value is about 0.07 dB/μm. As the wavelength range for DFWM varies, the pump wavelength should be tuned correspondingly. As a result, the chemical potential and the cross-sectional dimensions of the hybrid waveguide should also be optimized once more.

**Figure 4:**
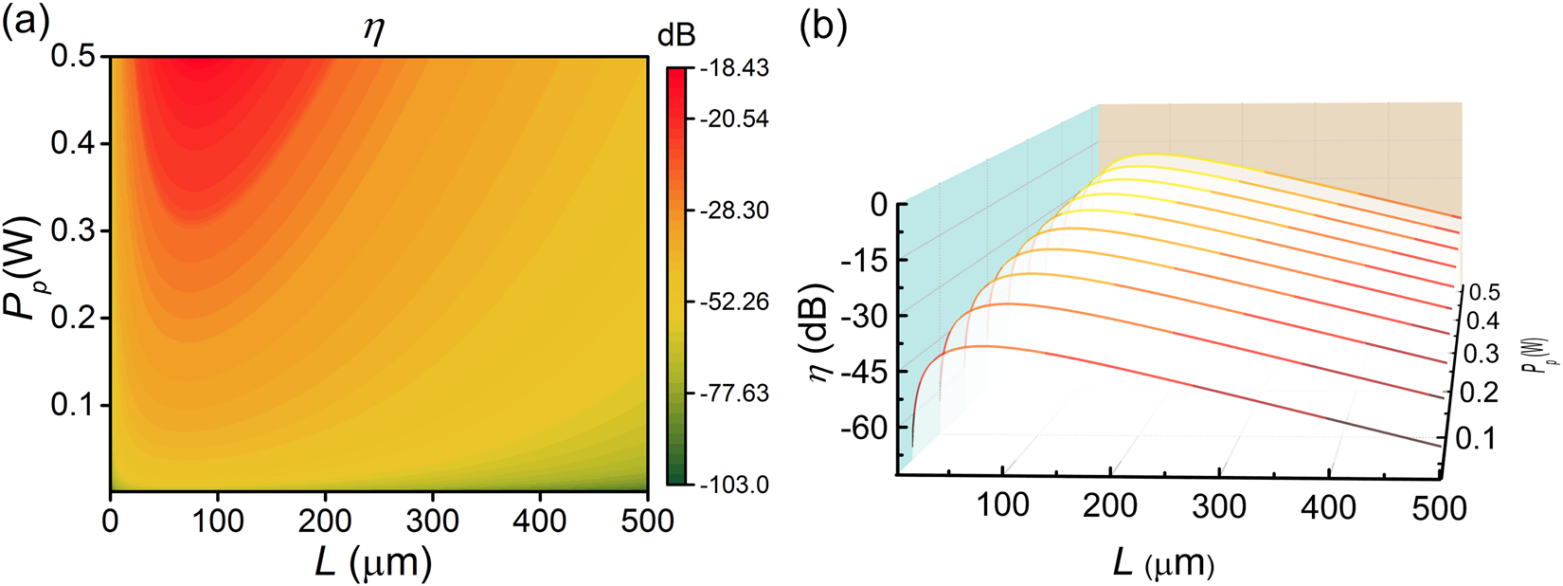
Optimization of the waveguide length *L* and the pump power *P*_*p*_. The optimal cross-sectional dimensions is 360 nm × 266 nm at the pump wavelength of 1.55 μm and graphene’s Fermi level is set at 0.4 eV. For a DFWM process, the pump and signal wavelength differential is 1 nm and the signal power is set to be 1 mW. (**a**) The conversion efficiency versus the waveguide length and the input pump power. (**b**) Waterfall of the horizontal cut lines in (**a**) with several pump powers.

Conversion efficiency of the DFWM process, which is defined as the ratio of the output idler power with respect to the incident signal power, is an important parameter which needs to be analyzed under various constrains. According to the coupled equations (11)–(13), the wavelength conversion ability of a specified waveguide structure mainly depends on two factors: the optical loss and the phase-matching condition. With the optimal cross-sectional dimensions (360 nm × 266 nm) at the pump wavelength of 1.55 μm, one can numerically solve these differential equations and achieve the maximum conversion efficiency by optimizing the input pump power and the waveguide length. Figure 4a shows the simulated conversion efficiency *η* versus the pump power (${P}_{p}$) and the waveguide length (*L*), where the signal wavelength ${\lambda }_{s}$ = 1.549 μm is set very closely to the pump wavelength. In this case, the linear phase mismatch is reasonable to be neglected. One can see that, for a given input pump power, the smooth conversion efficiency curve in Fig. 4b is hump-shaped along the propagating length and its maximum increases with the input pump power. The conversion efficiency will reach its maximum of −18.43 dB when the hybrid waveguide is 82 μm long and the pump power is 0.5 W. As the Fermi level of graphene ($|{\mu }_{c}|$ = 0.4 eV) is tuned to match the resonant condition at the pump wavelength, graphene exhibits the saturable absorption because the imaginary part of its nonlinear refractive index is negative from Fig. 1c. The estimated saturation field strength is about 3 × 10^7^ V/m^24^ which corresponds to about 1.2 W input light power in our case. For the validity of perturbation assumption of the whole theoretical model and avoid higher order nonlinearity in much tenser light intensity regime, we limit the pump power below 0.5 W in our numerical calculation.

**Figure 5:**
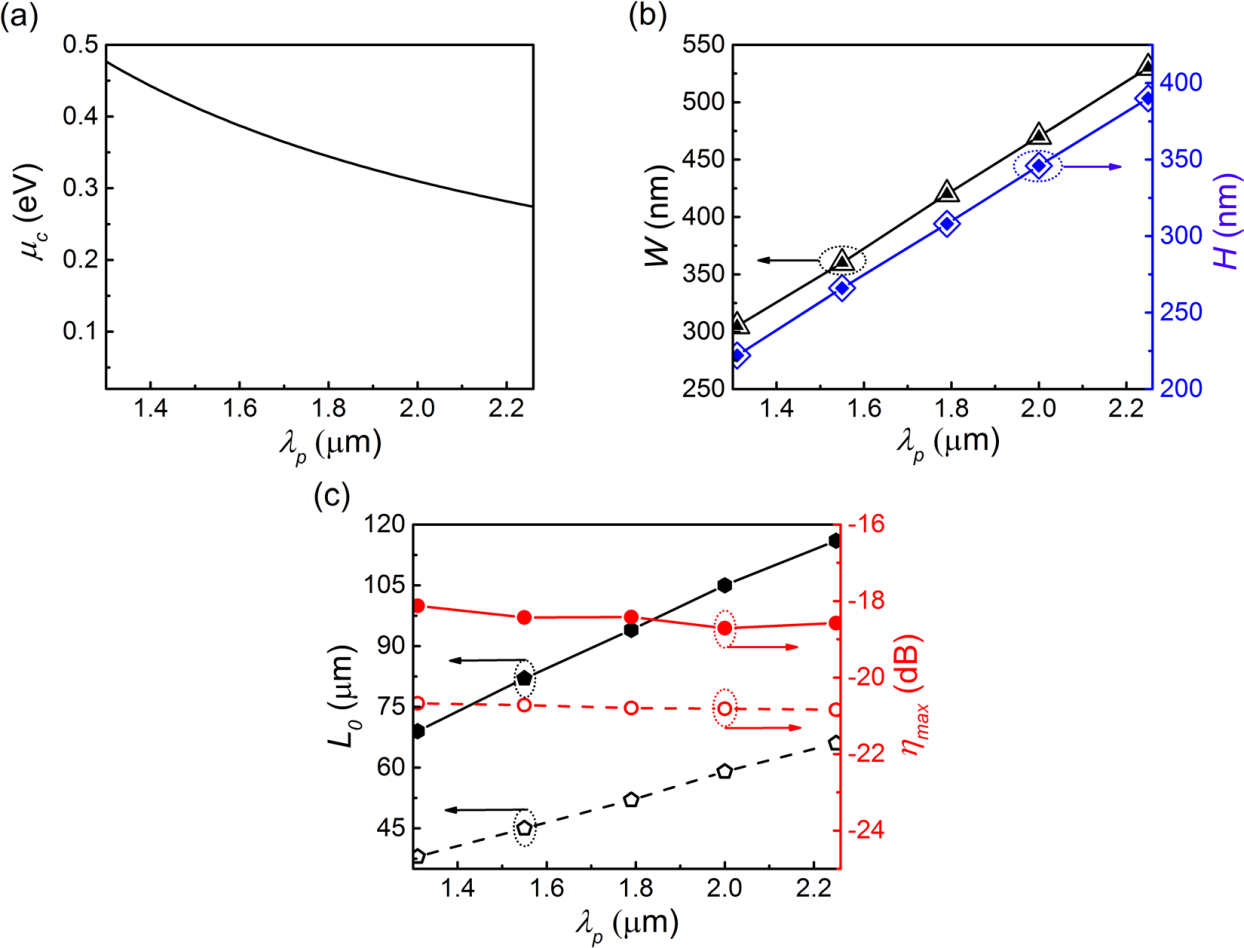
Geometry design in near- and mid-infrared spectrum. (**a**) The required chemical potential of graphene for different pump wavelength. (**b**) The optimal waveguide width *W* (triangle) and height *H* (diamond) as the pump wavelength varies. (**c**) The dark scatters mark the optimal waveguide length as the pump wavelength varies while the red ones are the corresponding maximum conversion efficiency.

Above the nonlinearity enhancement is demonstrated in near-infrared (telecommunication) band centering at 1.55 μm. Actually, according to the tunable low-loss bands of graphene and silicon, the designed graphene-silicon hybrid waveguide structure is expected to enhance the nonlinearity in a wide wavelength range, covering both near- and mid-infrared bands. For the applications in different bands, the pump wavelengths should be different, and the chemical potential of the graphene layer needs to be tuned to match the resonant condition ($\hslash {\omega }_{p}\approx 2|{\mu }_{c}|$) in Fig. 5a. Correspondingly, the cross-sectional dimensions and the waveguide length of the graphene-silicon hybrid waveguide also need to be optimized for various pump wavelengths. By using the similar procedures as above in 1.55 μm band, the optimized waveguide width *W* and height *H* are shown in Fig. 5b. One can find that the required waveguide width and height almost increase linearly with the working wavelength. In comparison, we also perform the cross-sectional-dimension optimization in the case of pristine graphene ($|{\mu }_{c}|$ = 0.02 eV) (hollow marks), and find that, for the same pump wavelength, the optimal dimensions are in agreement with the case of Fermi-level-tuned graphene (solid marks) as the marks in Fig. 5b of both cases overlap each other completely. Based on the DFWM process, the nonlinearity enhancement is evaluated with the optimal waveguide cross-section for each pump wavelength ${\lambda }_{p}$ by setting the wavelength spacing between the pump and the signal as 1 nm. Figure 5c shows the required waveguide length *L*_0_ and the maximum conversion efficiency that supported for the Fermi-level-tuned graphene (solid marks) and pristine graphene (hollow marks) cases when the pump and signal powers are 0.5 W and 1 mW, respectively. When the pump wavelength ${\lambda }_{p}$ varying from 1.31 μm to 2.25 μm, in both cases the optimal waveguide length *L*_0_ increases linearly and the corresponding maximum conversion efficiency ${\eta }_{\max }$ keeps in a high level, about −18.4 dB for the Fermi-level-tuned hybrid waveguide and −20.75 dB for the pristine-graphene one, and both fluctuates slightly in the range of 0.6 dB and 0.16 dB, respectively. Moreover, the conversion efficiency can be obviously improved by tuning the chemical potential of graphene. The maximum conversion efficiency of the Fermi-level-tuned hybrid waveguide is averagely 2-dB higher than that of the pristine-graphene hybrid waveguide since the propagation loss is reduced when the resonant condition of graphene is satisfied.

**Figure 6:**
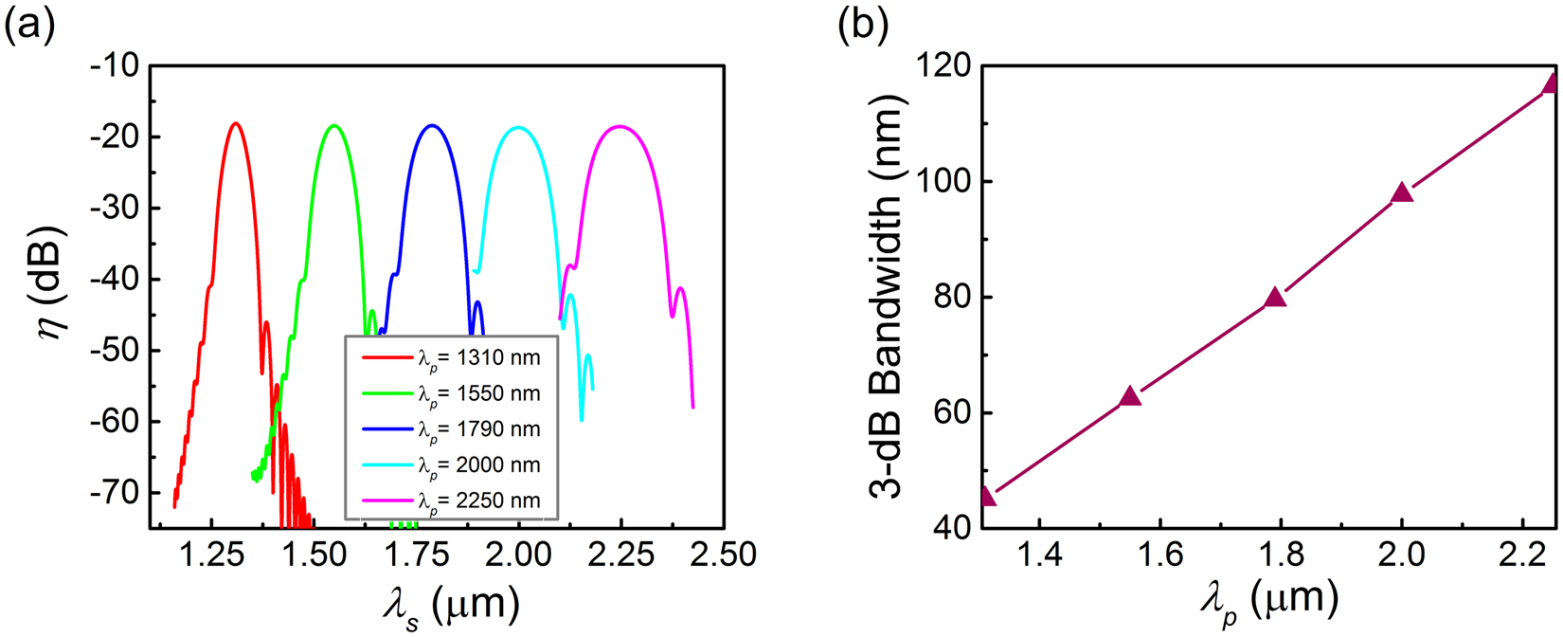
Simulation of the nonlinear performance of DFWM. The pump power and signal power are 0.5 W and 1 mW respectively. (**a**) Conversion curves at various pump wavelengths. (**b**) The 3-dB bandwidth.

Bandwidth performance is an important indication of the parametric conversion process in the graphene-silicon hybrid waveguide. According to the graphene’s Fermi level and the optimized waveguide cross-section dimensions for each pump wavelength, the conversion response for the DFWM process is simulated, as shown in Fig. 6a, where the conversion efficiency is calculated as the signal wavelength varies around the pump wavelength and the pump and signal powers are kept as 0.5 W and 1 mW. Here, a series of pump wavelengths in different bands are considered and the waveguide lengths are selected as their optimal values to support the maximum conversion efficiencies. The corresponding 3-dB bandwidth is shown in Fig. 6b as a function of the pump wavelength *λ*_*p*_. One can find that the DFWM in the graphene-silicon hybrid waveguide shows a broadband performance and the conversion bandwidth increases with the pump wavelength. In telecommunication band near 1.55 μm, a bandwidth of 62.5 nm can be obtained in an 82-μm-long 360 nm × 266 nm hybrid waveguide with the graphene’s chemical potential of 0.4 eV. While, in the mid-infrared 2 μm band, the conversion bandwidth reaches 97.7 nm, where a 105-μm-long 470 nm × 346 nm waveguide is needed and the graphene’s chemical potential need to be 0.31 eV. The results show that the designed graphene-silicon hybrid waveguide can support broadband nonlinearity enhancement in an ultra-broad wavelength range covering both near- and mid-infrared band.

In summary, a graphene-silicon hybrid waveguide with a dielectric spacer is proposed to enhance the nonlinearity using the high third-order susceptibility of graphene in an ultra-broad wavelength range covering near- and mid-infrared bands. The propagation loss of the hybrid waveguide is effectively reduced by tuning the graphene’s chemical potential to meet the resonance condition, and the dielectric spacer blocks the free-carrier exchange to avoid the additional loss. A full-vectorial theoretical model is established to analyze the nonlinear DFWM effect in the proposed hybrid waveguide. The waveguide’s cross-sectional dimensions are optimized in terms of the nonlinear parameter and the working wavelength. With a 0.5 W input pump power, the maximum conversion efficiency in the Fermi-level-tuned hybrid waveguide with optimal geometry reaches about −18.5 dB and the required waveguide length is only tens of microns. The conversion bandwidth is as broad as 62.5 nm in 1.55 μm band, and it reaches 97.7 nm in 2 μm band. The proposed hybrid waveguide structure has a stable nonlinear enhancement from 1.3–2.3 μm, which exhibits that the micron-scale graphene-silicon hybrid waveguide has the potential to support chip-scale nonlinear applications in both near- and mid-infrared bands.

## Methods

By considering the graphene layer as a thin film with thickness of 0.5 nm^33^, its linear conductivity ${\sigma }_{g}^{(1)}$ is obtained from the Kubo formula^32^ and the third-order conductivity ${\sigma }_{g}^{(3)}({\omega }_{1},\,{\omega }_{2},\,{\omega }_{3})$ is calculated based on Mikhailov’s theory^27^. The simulation results of the effective mode index and the field distribution are found by using the two-dimensional finite-element-frequency-domain (FEFD) method. The coupled equations (11)–(13) is obtained using the reciprocal theorem^36^ and solved by a fourth order Runge-Kutta method with a length step Δ*z* = 0.3 μm. The simulation parameters have been given in our paper.

### Data availability

All data generated or analyzed during this study are included in this article.
